# Influence of cell culture media and feed supplements on cell metabolism and quality of IgG produced in CHO-K1, CHO-S, and CHO-DG44

**DOI:** 10.1186/1753-6561-9-S9-P36

**Published:** 2015-12-14

**Authors:** David Reinhart, Lukas Damjanovic, Wolfgang Sommeregger, Andreas Gili, Stanislaus Schafellner, Andreas Castan, Christian Kaisermayer, Renate Kunert

**Affiliations:** 1 Department of Biotechnology, University of Natural Resources and Life Sciences, Vienna, Muthgasse 11, 1190 Vienna, Austria; 2 Polymun Scientific Immunbiologische Forschung GmbH, Donaustraße 99, 3400 Klosterneuburg, Austria; 3 GE Healthcare Bio-Sciences AB, Björkgatan 30, 75184 Uppsala, Sweden; 4 BioMarin International Limited, Shanbally, Ringaskiddy, County Cork, Ireland

## Background

Chinese hamster ovary (CHO) cells have become the preferred expression system for the production of complex recombinant proteins. In this study, chemically defined CHO cell culture media and feed substrates from different vendors were investigated regarding their influence on cell metabolism, antibody titer and quality. Special emphasis was put on elucidating how these attributes change with the use of different CHO host cell lines. For this purpose, CHO-K1, CHO-DG44, and CHO-S - each producing the same IgG antibody - were adapted to ActiCHO™ P and CD CHO medium. All three cell lines were grown both in batch and in fed-batch cultures using the manufacturer's specific concentrated feed supplements. The impact of the different media and feeds on antibody production, cell growth, cell-specific nutrient consumption, by-product formation and IgG quality was analyzed throughout the process.

## Materials and methods

CHO-K1, CHO-S and CHO-DG44 cell lines producing the same IgG were grown in two different cultivation media, ActiCHO P (GE Healthcare) and CD CHO (Life Technologies). Batch and fed-batches were performed in Erlenmeyer shake flasks (Corning, NY) in a CO2 incubator shaker (Kühner, Switzerland) at 37°C, 7% CO2, 140 rpm and 90% relative humidity. All experiments were performed in triplicates.

Fedbatches were fed with the corresponding feeds ActiCHO Feed A and Feed B (GE Healthcare), EfficientFeed™ A and FunctionMAX™ (both Life Technologies) according to the manufacturer's instructions [1]. The respective feeding regimens are shown in Table 1. The residual glucose concentration was maintained above 3 g/L by addition of glucose concentrate.

**Table 1:** Fedbatch feeding strategies.

Basal medium	ActiCHO Feed A	ActiCHO Feed B	EfficientFeed A	FunctionMAX
ActiCHO P	Daily; 3%	Daily; 0.3%	-	-
CD CHO	-	-	4, 6, 8; 10%	4, 6, 8; 3.3%

The time [d] for feed addition and the feed volume in % of the culture volume are indicated. Feed start for the culture in ActiCHO P was day 3.

Daily, samples were drawn to determine of cell concentration, viability, IgG titer, metabolites (i.e. glucose, glutamine, glutamate, lactate, ammonium), osmolality. Additionally, on day 4 (mid-exponential growth phase), day 7 (end exponential growth phase) and at culture termination (=viability <60%) we analyzed residual amino acid concentrations, gene copy numbers and mRNA, intracellular product content by flow cytometry, as well as antibody quality by SDS-PAGE, Western blotting, glycosylation, product aggregation, distribution of charge variants and thermal stability.

## Results

In general, fedbatch strategies considerably prolonged the process duration as compared to the batch cultures. As a consequence, higher cell peak concentrations were obtained which additionally boosted the antibody titers up to more than six-fold (data not shown).

**Figure 1:**
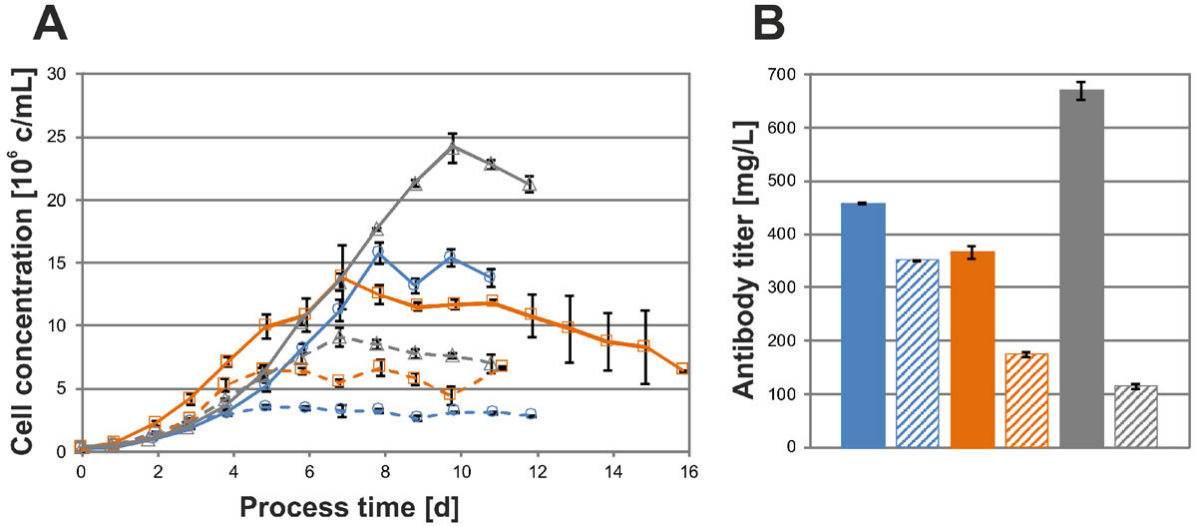
**(A) Cell concentrations and (B) harvested antibody titer during fedbatch cultivations**. CHO-K1 (blue), CHO-S (orange) and CHO-DG44 (grey) grown in ActiCHO P are shown in continuous lines or full bars, whereas cultures in CD CHO are shown in dashed lines or patterned bars.

As shown in Figure 1, the highest cell and antibody concentrations were obtained when ActiCHO P was used as basal medium for all three recombinant cell lines (CHO-K1, CHO-DG44 and CHO-S). The higher specific growth rate in ActiCHO P resulted in higher peak cell concentrations and an up to five-fold difference in the viable cell integral (data not shown). Up to six-fold differences in IgG titers were found between cells grown in the two media (Figure 1B). The specific rates of IgG production were in good agreement with the intracellular expression of light and heavy chain mRNA levels in all three CHO cell lines (data not shown). Consequently, the higher viable cell integral in ActiCHO P fed-batches was the main contributor to the higher antibody concentrations.

The two fed-batch strategies clearly influenced the cell-metabolic rates of nutrient consumption and by-product formation (data not shown). For example, the cell-specific glucose consumption rates were 30-55% higher in CD CHO medium for all three CHO cell lines. Further, the three CHO cell lines converted glucose to lactate with different efficiencies. Depending on the host cell line 10-15% (CHO-S), 25% (CHO-DG44) and 40% CHO-K1 of glucose was converted to lactate. Glutamine, a major energy source for CHO cells, was consumed at comparable cell-specific rates in both media. Moreover, the cell-specific rates of glutamine consumption were determined to be highly host cell line-specific. Another remarkable finding was that all cell lines revealed a net consumption of glutamine in CD CHO fed-batches, whereas a net production was observed in ActiCHO P cultures. This finding may influence the design of future feeding strategies. The specific rates of ammonium production were not altered by the medium for CHO-K1. However, 30-60% lower values were obtained when CHO-S and CHO-DG44 were grown in ActiCHO P fed-batches.

The harvested cell culture supernatant of all triplicate fed-batch cultures was purified and subsequently analyzed for antibody quality by different techniques. All samples appeared homogeneous, pure and of correct molecular weight on an SDS-PAGE and the amount of aggregated product was less than 5% as determined by SEC (data not shown).

Glycosylation of the antibody's Fc domain was alternated in response to the usage of CHO cell line as well as feeding strategy (data not shown). Generally, higher levels of fucosylation were determined for CHO-S cultures as well as fed-batches in CD CHO. IgG produced by CHO-S and CHO-K1 had higher levels of galactosylation when cultivated in ActiCHO P, whereas the CHO-DG44 cell line was not affected by the feeding strategy. Mannose structures were most efficiently processed (=lowest high-mannose structures) in CHO-S, followed by CHO-K1 and CHO-DG44.

## Conclusions

The medium highly affected the bioproduction process and yielded in up to five-fold higher peak cell and in up to six-fold higher antibody titers. Also the cell-metabolic rates (i.e. glucose and glutamate) were influenced by the production medium. Lactate production and cell-specific rates of glutamine consumption highly depended on the CHO host cell line. Regarding product quality, the choice of medium and CHO host cell line both can be used to fine-tune antibody glycosylation such as fucosylation, galactosylation and mannosylation.

## Acknowledgements

We thank GE Healthcare and Polymun Scientific ImmunbiologischeForschung for funding this study.

## References

[B1] Barrett S Boniface R Dhulipala P Slade P Tennico Y Stramaglia M Lio P Gorfien SF Attaining next-level titers in CHO fed-batch cultures BioProcess International 2012 10 56 62

